# Immediate analgesic effect of two modes of transcutaneous electrical nerve stimulation on patients with chronic low back pain: a randomized controlled trial

**DOI:** 10.31744/einstein_journal/2021AO6027

**Published:** 2021-12-03

**Authors:** Madeline Luiza Ferreira Pivovarsky, Fernanda Gaideski, Rafael Michel de Macedo, Raciele Ivandra Guarda Korelo, Luiz César Guarita-Souza, Richard Eloin Liebano, Ana Carolina Brandt de Macedo

**Affiliations:** 1 Universidade Federal do Paraná Curitiba PR Brazil Universidade Federal do Paraná, Curitiba, PR, Brazil.; 2 Pontifícia Universidade Católica do Paraná Curitiba PR Brazil Pontifícia Universidade Católica do Paraná, Curitiba, PR, Brazil.; 3 Universidade Federal de São Carlos São Carlos SP Brazil Universidade Federal de São Carlos, São Carlos, SP, Brazil.

**Keywords:** Transcutaneous electric nerve stimulation, Low back pain, Pain measurement, Chronic pain, Physical therapy modalities

## Abstract

**Objective:**

To compare and assess the immediate analgesic effects of conventional and burst transcutaneous electrical nerve stimulation in patients with chronic low back pain.

**Methods:**

We conducted a three-arm single-blinded randomized controlled trial. A total of 105 patients with non-specific chronic low back pain aged between 18 and 85 years were randomly assigned into the following groups: Placebo Group (sham electrical stimulation), Conventional TENS Group (continuous stimulation at 100Hz for 100µs with sensory intensity), and Burst TENS Group (stimulation at 100Hz modulated at 2Hz for 100µs with motor-level intensity). All groups received a single application of transcutaneous electrical nerve stimulation for 30 minutes. The outcomes, namely, pain intensity, quality of pain, and pressure pain threshold were measured by the visual analog scale, McGill pain questionnaire, and algometry, respectively. The patients were evaluated before and immediately after the transcutaneous electrical nerve stimulation application.

**Results:**

Pain intensity (visual analog scale score) and quality of pain (McGill pain questionnaire score) significantly decreased (p<0.05) in Intervention Groups (Conventional TENS Group and Burst TENS Group). A positive effect was observed in the interventions compared to the Placebo Group in all domains of the McGill pain questionnaire (p<0.05), excepting for the pain intensity. Pressure pain threshold significantly increased (p<0.05) immediately after the transcutaneous electrical nerve stimulation application in both Intervention Groups, but not in the Placebo Group. For significant difference was found during assessment when comparing both Intervetion Group.

**Conclusion:**

Both transcutaneous electrical nerve stimulation modes were effective for pain modulation. Moreover, there was an increase in the pressure pain threshold. No significant results were found to indicate the best mode for the treatment of chronic low back pain.

**Clinical Trial Registration:** RBR-59YGRB.

## INTRODUCTION

The orthopaedic section of the American Physical Therapy Association defined chronic low back pain (CLBP) as generalized low back pain, which lasts longer than 3 consecutive months.^(1)^ The notion of non-specific CLBP is often used to describe this condition because the mechanism of pain is poorly understood. The clinical practice guidelines^(2)^ define CLBP as the absence of red flag symptoms for more serious causes of pain with multifactorial pathogenesis.

According to the Brazilian Statistical Yearbook of Work-Related Accidents,^(3)^ approximately 34,253 cases of back pain (CID10 M54) were registered in Brazil in 2013, being low back pain (LBP) ranked as the fourth common injury. Low back pain was the most frequent type of back pain, and it was one of the main causes of absence from work. Moreover, a systematic review on the prevalence of LBP in Brazil reported high 1-year prevalence rate (>50%) among adults.^(4)^

Low back pain overloads all health services and various interventions have been established to treat this such as medication, surgery, patient education, behavioral therapy, and physiotherapy.^(2)^ Among the possible physical therapy treatments, electrophysical agents as the transcutaneous electrical nerve stimulation (TENS) are widely used for the management of CLBP as a complement to other therapeutic interventions, particularly for exercising. Transcutaneous electrical nerve stimulation is relatively safe, non-invasive, easy to apply,^(5)^ and it provides pain relief during exercise.^(2)^ Different TENS modalities differing in frequency, amplitude, pulse duration, and waveform can be used in clinical practice, such as the conventional or burst modes.

Studies have reported that TENS can reduce acute and chronic pain of different etiologies,^(2,6-10)^ however, the optimal parameters in CLBP treatment are still unknown.^(5)^ When TENS is applied with high frequency, such as in the conventional mode, the physiological intention is to produce Aβ fiber depolarization effect, capable of inhibit transmission of nociceptive information for spinal cord.^(6)^ When TENS is applied at low frequency and strong stimuli, such as burst mode, the physiological intention is depolarizes the fast pain (Aδ) fibers capable to activation of descending analgesia.^(10)^ In addition, both TENS activate opioid receptors in the central nervous system, which induces analgesia *e.g.,* such serotonin and Mμ or delta opioid receptors, when low or high frequency TENS is applied, respectively.^(7,10)^

Despite the extensive use, there is still no consensus on the actual effectiveness of TENS to individuals with LBP.^(8)^ A systematic review^(8)^ on TENS efficacy in CLBP was conducted in four high-quality randomized controlled clinical trials (585 patients) wherein only three showed pain relief compared to the Placebo Group (PG). Due to the small number of studies, it was not possible to find supporting evidence on the efficacy of this procedure in patients with LBP. Hence, no evidence on the appropriate parameters and stimulation modes was found. Jauregui et al.,^(9)^ conducted a meta-analysis and concluded that treatment using TENS resulted in not only reduced pain, but also reduced drug intake among patients with CLBP treated for less than 5 months.

Johnson et al.,^(10)^ evaluated TENS use in the management of acute pain caused by multiple etiologies wherein 19 studies were reviewed (1,346 patients). The authors were able to assess the analgesic effect of TENS in six studies and found that TENS was 3 times more effective than the placebo. However, they found low to moderate evidence due to the small number of patients and high risk of biased results. An editorial by Johnson et al.,^(11)^ suggested that most studies on the use of TENS were inconclusive and of insufficient quality. In addition, a meta-analysis by Resende et al.,^(5)^ reported the necessity of more quality studies on the effects of TENS on CLBP patients considering factors, such as stimulation intensity, pain, incapacity and functionality assessment, and time for evaluating results.

All systematic reviews^(8-10)^ pointed to studies comparing a single TENS parameter with PG or with other therapies. Of the studies cited in these reviews, only one^(12)^ compared three types of TENS (conventional TENS, acupuncture TENS, biphasic TENS and PG), and during one month of treatment no differences were found between groups. However, this study did not describe the exact parameters of application. No studies have compared different parameters of TENS application in CLBP

Although there are several studies on the effect of TENS on LBP,^(13,14)^ most of them only report the long-term effects. However, the main therapeutic goal during a physical therapy session is an immediate analgesic effect-either to enable patients to practice physical exercises or to relieve pain after a therapy session.

## OBJECTIVE

To compare and assess the immediate analgesic effects of conventional and burst transcutaneous electrical nerve stimulation in patients with chronic low back pain.

## METHODS

### Study design

A three-arm single-blinded randomized controlled trial was conducted. The study was approved by the Research Ethics Committee, CAAE: 44642615.2.0000.0102, number 1.145.540, and prospectively registered on ensaiosclinicos.gov.br (RBR-59YGR8). All patients signed a consent form before the study begins.

### Study location

The present study was conducted in the *Universidade Federal do Paraná* (UFPR) physical therapy laboratory and ambulatory of the *Hospital das Clínicas,* Curitiba, PR, Brazil.

### Eligibility criteria

The inclusion criteria included patients aging 18-85 years with a primary diagnosis consistent with non-specific LBP (LBP not otherwise specified that lasted more than 3 months)^(2)^ without irradiation, and with a minimum pain intensity of three on the Numeric Pain Rating Scale (NPRS).^(15)^

The exclusion criteria were as follows patients with specific causes of pain (*e.g*., disc herniation, spinal stenosis, vertebral fracture, infection, back-related tumor, osteoporosis, rheumatoid arthritis, inflammatory processes, pregnancy, and kidney problems); previous surgery in the lumbar region; skin lesions; presence of pacemaker; using medications for relief from LBP 48h before our intervention; and those currently undergoing some treatment for the LBP.

### Randomization and interventions

After the baseline assessment, the participants were randomized using random blocks.^(16)^ Each block contained 15 participants randomly distributed, that is, PG (n=5), Conventional TENS Group (CTG) (n=5), and Burst TENS Group (BTG) (n=5). A total of 7 blocks were inserted including 105 participants that were blinded to the intervention. The intervention was conducted by one physiotherapist who was unaware of the object of the study.

Electrical stimulation was applied using the same device (TENSys ET 9771 equipment, KLD Biosistemas Electronics, São Paulo, SP, Brazil) previously calibrated by the MedMart (Brazil). The participants were placed in a prone position and instructed to relax the musculature while the procedures were performed. Four rectangular surface (90x50mm) electrodes were bilaterally placed at the level of the L3 and L5 spinous process (30mm laterally). With this quadripolar placement, the current in each channel crossed the area experiencing the pain. After cleansing the skin with rubbing alcohol 70%, carbon-impregnated silicone rubber, and flexible electrodes were placed using an electroconductive gel and fixed with an adhesive tape.^(17)^ The CTG was administered using a continuous stimulation (biphasic, rectangular, symmetrical and balanced waveform) at a high frequency (100Hz) with pulse duration (100µs), and sensory intensity. The BTG (acupuncture-like TENS was administered in the BTG using a 100Hz modulated at 2Hz (burst mode) and a pulse duration of 100μs with motor-level intensity. Transcutaneous electrical nerve stimulation was applied only once for 30 minutes, and reassessed was performed immediately following the intervention. The interval between assessment and reassessment was 30 minutes. The procedures for the PG were similar to those of the other groups. However, the current amplitude/intensity did not increase. Participants were informed that they may or may not experience any sensation on the site of application of the electrodes.^(18)^

### Evaluation and instruments

One blinded trained physiotherapist examined all the participants prior to and immediately after the intervention. The measured outcomes were pain intensity, quality of pain, and pressure pain threshold (PPT).

### Pain intensity measurements with the Numerical Pain Rating Scale

The participants were instructed to point out what best represented the intensity of their pain using a 10cm straight line on the NPRS (0=no pain, 10=worst pain ever).^(19)^

### Quality of pain measurements with the McGill Pain Questionnaire

The Brazilian-Portuguese Long Form^(20)^ of the McGill pain questionnaire (MPQ) contains 78 descriptors divided into 4 subclasses of pain quality (sensory, affective, cognitive, and miscellaneous). The descriptors were divided into 20 subcategories, each containing 4 to 6 words. Patients had to choose one or none of the words in all subcategories, and the numeric index was calculated by summing the number of chosen options, with a maximum value of 20. The Brazilian-Portuguese Long Form-MPQ was cross-culturally adapted to Brazilian Portuguese^(18)^ and clinimetrically tested in a previous study.^(21)^

### Pressure pain threshold measurements with algometry

The PPT was measured using an algometer (EMG system, São José dos Campos, SP, Brazil) with the tip measuring 1cm^2^ in diameter and with an application rate of 0.5kgf/cm^2^/s.

Pressure algometry results have previously shown good validity.^(22)^ However, in our study, the single blinded physiotherapist conducted a preliminary intra-observer reliability evaluation. She evaluated 10 individuals to assess the intra-test reliability, with an interval of at least 48 hours. The intra-test (ICC) analysis indicated excellent reliability for the intervention results (ICC=0.99).

The PPT was measured bilaterally at the following points^(18)^ previous marked with a dermatographic pencil: 5cm to the left and right spinous process of the L3 and L5 vertebra. Two more points were used as a control and marked 5cm lateral to the tibial tuberosity in the left and right tibialis anterior muscle. After marking the points, the tip of the algometer was positioned perpendicular to the skin and gradually pressed at a rate of 0.5kgf/cm^2^/s, starting with zero kgf/cm^2^/s. The participants were instructed to say “stop” when the pressure of the algometer’s rubber tip caused pain. Then, this value was registered by the researchers and represented the PPT at the time. After 1 minute, two more measurements were performed at each point, and the average was calculated for each point.

### Statistical analysis

All statistical analyses were performed using the SPSS 25.0. Data were presented as mean±standard deviation of the mean and they were subjected to analysis of the sphericity and homogeneity of variance using the Mauchly’s and Levene’s tests, respectively. The confidence interval was set at 95% for all analyses. To analyze the differences between and within groups, analysis of variance with repeated measures was used for parametric values. For non-parametric data, the Wilcoxon signed-rank test was used to verify the difference within groups and the Kruskal-Wallis test was used to establish the differences between groups. The level of significance was set at p<0.05.

### Sample size calculation

The program GPower 3.0 was used to calculate the sample size. The statistical power was set at 0.95, a moderate effect size (0.35),^(23)^ a statistical power of 95% (1β error probability), an α error level probability of 0.05, and a possible sample loss of up to 5% were considered. Considering these calculations, we included 35 patients per group (105 in total).

## RESULTS

**Figure 1:**
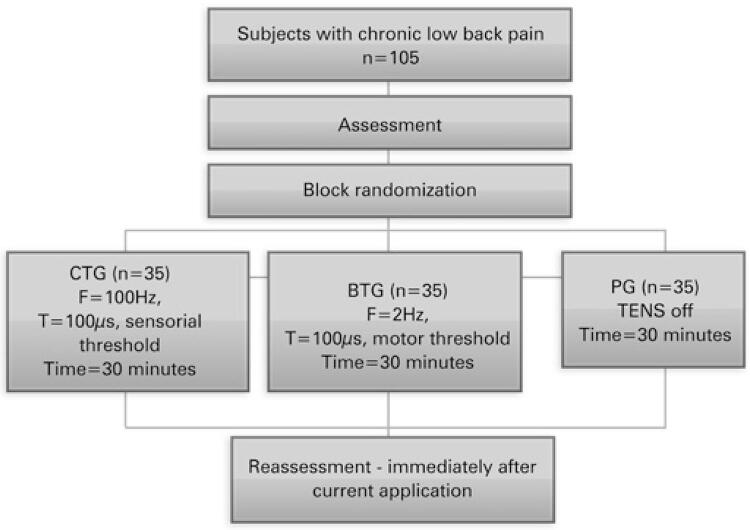
Study design F: frequency; T: time of pulse width; CTG: Conventional TENS Group; BTG: Burst TENS Group; PG: Placebo Group.

A total of 105 patients were evaluated from August 2016 to August 2017. No participants left the study before its completion. The consolidated standards of reporting trials diagram is shown in figure 1. The socio-demographic characteristics are presented in table 1.

**Table 1:** Socio-demographic characteristics

Socio-demographic characteristics	PG (n=35)	CTG (n=35)	BTG (n=35)	p value
Age [mean (SD)]	40.8±2.7	44±2.2	42.6±2.2	0.44
Gender, n (%)				0.29
Female	24 (68.6)	23 (65.7)	27 (77.1)	
Male	11 (31.4)	12 (34.3)	8 (22.9)	
Formal education, n (%)				0.41
Incomplete primary school	6 (17.1)	3 (8.6)	7 (20)	
Complete primary school	2 (5.7)	2 (5.7)	4 (11.4)	
Incomplete high school	2 (5.7)	2 (5.7)	4 (11.4)	
Complete high school	10 (28.6)	6 (17.1)	6 (17.1)	
Incomplete college	5 (14.3)	5 (14.3)	4 (11.4)	
Complete college	10 (28.6)	17 (48.6)	10 (28.6)	
Life habits, n (%)				
Smoking	4 (11.4)	4 (11.4)	2 (5.7)	0.42
Alcohol consumption	1 (2.9)	0 (0)	1 (2.9)	0.38
Sedentary	27 (77.1)	22 (62.9)	23 (65.7)	0.25
Time of pain in years (mean, minimum, maximum, median)	6.81; 0.5; 30; 5	8.8; 1; 30; 7	7.25; 0.5; 30; 3	0.31
Pain localization, n (%)				0.94
Centralized	7 (20)	9 (25.7)	9 (25.7)	
Right side	7 (20)	6 (17.1)	7 (20)	
Left side	6 (17.1)	8 (22.9)	6 (17.1)	
Bilateral	15 (42.9)	12 (34.3)	13 (37.1)	
Period of worsening of pain, n (%)				0.06
Morning	9 (25.7)	11 (31.4)	4 (11.4)	
Afternoon	11 (31.4)	7 (10)	19 (54.3)	
Night	15 (42.9)	17 (48.6)	12 (34.3)	
Activities that worsen the pain, n (%)				
Walk	11 (31.4)	11 (31.4)	14 (40)	0.75
Sit	8 (22.9)	10 (28.6)	11 (31.4)	0.79
Bend	19 (54.3)	21 (60)	15 (42.9)	0.15
Get up	13 (37.1)	12 (34.3)	11 (31.4)	0.80
Climb stair	11 (31.4)	7 (20)	6 (17.1)	0.77
Effort/Lift object	29 (82.9)	31 (88.6)	29 (82.9)	0.51

PG: Placebo Group; CTG: Conventional TENS Group; BTG: Burst TENS Group; SD: standard deviation.

Table 2 shows the mean and standard deviation of pain intensity NPRS and quality of pain (MPQ score). A statistical difference was noted for parameters within the groups.

**Table 2:** Mean (standard deviation) at baseline (before) and 30 minutes (after) follow-up for subjects with chronic low back pain who received conventional transcutaneous electrical nerve stimulation, burst transcutaneous electrical nerve stimulation or Placebo Group

Outcome	Interventions					

	CTG (n=35) Mean (DP)		BTG (n=35) Mean (DP)		PG (n=35) Mean (DP)	
		
	Before	After	Before	After	Before	After
Pain (0-10)	5.4 (1.6)	2.3 (2.1)*	4.7 (2.2)	2.4 (2.1)*	4.2 (2.2)	3.0 (2.4)*
MPQ						
Sensory	7.1 (2.2)	3.8 (3.4)*	7.8 (2.3)	3.4 (3.4)*	7.5 (2.4)	4.6 (3.4)*
Affective	3.0 (1.8)	0.6 (1.3)*	3.7 (1.4)	1.1 (1.5)*	3.3 (1.3)	1.6 (1.6)*
Cognitive	1.0 (0.1)	0.5 (0.5)*	1.0 (0)	0.4 (0.5)*	0.9 (0.1)	0.6 (0.4)*
Miscellaneous	2.5 (1.1)	1.3 (1.4)*	3.0 (0.8)	1.4 (1.5)*	2.8 (1.3)	1.9 (1.5)*
Total	13.5 (5.1)	6.3 (5.8)*	15.5 (4.1)	6.6 (6.3)*	14.5 (5.1)	8.8 (6.2)*

* p<0.05 within groups.

DP: standard deviation; PG: Placebo Group; CTG: Conventional TENS Group; BTG: Burst TENS Group; MPQ: McGill Pain Questionnaire.

**Figure 2:**
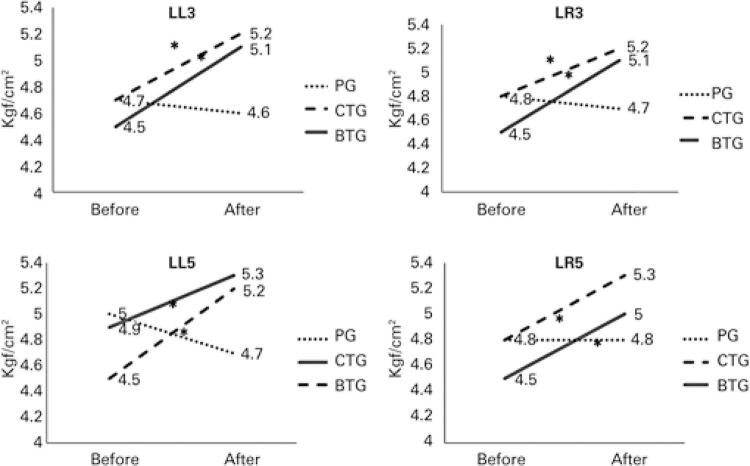
Pressure pain threshold at baseline (before) and 30 minutes (after) follow-up for subjects with chronic low back pain who received conventional transcutaneous electrical stimulation and burst transcutaneous electrical stimulation or Placebo Group * p<0.05 within groups. LL3: third left lumbar vertebra; LR3: third right lumbar vertebra; LL5: fifth left lumbar vertebra; LR5: fifth right lumbar vertebra; PG: Placebo Group; CTG: Conventional TENS Group; BTG: Burst TENS Group.

Figure 2 shows the PPT results. The TENS Groups showed a significant increase in PPT (p<0.05) in all lumbar points on pressure algometry. However, a non-significant decrease was noted in the PG.

Table 3 shows the between-group analysis for all comparisons. The statistical analysis showed no differences between Interventions Groups (CTG and BTG) using the Visual Analogue Scale (VAS) compared to placebo. In addition, while a 28.5% decrease in pain intensity was of noted in the PG, a 30 minutes intervention led to a 57.4% and 48.9% decrease in pain intensity in the CTG and BTG, respectively. A statistically significant difference was observed between both TENS Groups and PG based on the MPQ subcategories (sensory, affective, cognitive and miscellaneous), and numeric index. In the MPQ, CTG showed a 53.3% increase in numeric index, BTG 57.4% while PG only 39.3%. However, no significant difference was found when comparing both CTG and BTG. On pressure algometry, no statistical difference was found comparing CTG and BTG to PG, although an increase in the pressure pain threshold was found in the Intervention Groups, and a decrease in placebo.

**Table 3:** Between-group differences at 30 minutes follow-up after randomization for subjects with chronic low back pain who received conventional transcutaneous electrical nerve stimulation, burst transcutaneous electrical nerve stimulation, or Placebo Group

Outcome	CTG *versus* Placebo	p value	BTG *versus* Placebo	p value	CTG *versus* BTG	p value
Pain (0-10)	0.1 (-0.9-1.2)	1.00	0.2 (-0.9-1.2)	1.00	0.3 (-0.7-1.4)	1.00
MPQ						
Sensory (0-10)	2.1 (1.1-3.1)	0.00*	1.9 (0.9-2.9)	0.00*	0.2 (-0.7-1.1)	1.00
Affective (0-5)	0.9 (0.3-1.4)	0.00*	0.8 (0.2-1.4)	0.02*	-0.1 (-0.4-0.6)	1.00
Cognitive (0-1)	0.2 (0.1-0.3)	0.00*	0.2 (0.1-0.3)	0.00*	1.8 (-013-0.1)	1.00
Miscellaneous @(0-4)	1.0 (0.5-1.4)	0.00*	0.9 (0.4-1.3)	0.00*	-0.8 (-0.3-0.5)	0.18
Total (0-20)	1.7 (3.8-1.4)	0.03*	-0.5 (3.2-2.0)	0.02*	-1.1 (-1.5-3.7)	0.80
Algometry						
LTA	-0.9 (-1.1-0.9)	1.00	-0.1 (-1.1-0.9)	1.00	-0.2 (-1.2-0.9)	1.00
RTA	0.0 (-1.1-1.1)	1.00	-0.1 (-1.2-0.9)	1.00	-0.15 (-1.2-0.9)	1.00
LL3	0.2 (-0.7-1.2)	1.00	0.0 (-0.9-0.0)	1.00	-0.2 (-1.1-0.7)	1.00
RL3	0.2 (-0.7-0.5)	1.00	0.0 (-0.9-0.9)	1.00	0.2 (-1.1-0.7)	1.00
LL5	0.2 (-0.8-1.2)	1.00	0.0 (-1.0-1.0)	1.00	-0.2 (-1.2-0.8)	1.00
RL5	0.2 (-0.7-1.2)	1.00	-0.1 (-1.0-0.8)	1.00	-0.3 (-1.3-0.6)	1.00

* Significant difference (p<0.05). Adjusted mean difference (95%CI); Baseline to follow-up at 30 minutes (95%CI).

95%CI: 95% confidencial interval; RTA: right of tibialis anterior muscle; LTA: left of tibialis anterior muscle; LL3: left of third lumbar vertebrae; RL3: right of third lumbar vertebrae; LL5: left of fifth lumbar vertebrae; RL5: right of fifth lumbar vertebrae; CTG: Conventional TENS Group; BTG: Burst TENS Group; MPQ: McGill Pain Questionnaire.

## DISCUSSION

This study aimed to assess the immediate analgesic effects of Conventional and Burst TENS modes in patients with CLBP, which is assessed by using the NPRS, MPQ, and pressure algometry.

To the best of our knowledge, this is the first study which compares the immediate analgesic effect of two different modes of TENS in CLBP. Immediate pain relief is necessary to enable patients to perform rehabilitation exercises, as pain can be a limiting factor or intense pain that may occur following a therapy session.

In the present study, no difference in NPRS results was identified between the TENS Groups. Thus, Conventional and Burst TENS modes were equally effective in providing immediate pain relief. High- and low-frequency TENS (conventional) promote analgesia through the release of endogenous opioids at the spinal level and rostral ventral medulla. The different stimulation frequencies led to the release of different opioids, *i.e*., high frequencies (more than 50Hz) stimulate δ-opioid receptors and low frequencies (less than 10Hz) activate µ-opioid receptors.^(24)^ Since the analgesic pathways are not the same, it was expected that the responses would also be different. However, the difference of responses was not noted.^(9)^ This data corroborates with a study conducted by Cheing et al.,^(25)^ wherein TENS (80Hz, 140μs, 60 minutes) was applied in individuals with chronic pain and a decrease in the pain pattern that was verified through the VAS score after a single application. Tousignant-Laflamme et al.,^(26)^ compared the outcomes of 1^st^ round of TENS acupuncture applied for different time periods (15 minutes and 30 minutes) in 11 patients with CLBP and found no difference in the VAS score between the groups. However, no comparison with the PG was performed.

In the assessment by pressure algometry, positive results were observed, with an increase in the PPT ranging from 8% to 15% in groups treated with TENS compared with a 2% to 5% reduction in the PG. Ebadi et al.,^(1)^ evaluated the analgesic effects of TENS (120Hz, 100µs, 15 minutes) and dynamic currents on the PPT of patients with CLBP and found an increase in PPT in the TENS Group. In a trial conducted by Giesbrecht et al.,^(27)^ the PPT in LBP patients and healthy individuals was compared. The results revealed that the Group with LBP had a lower PPT, which indicates local sensitization. According to the study, central sensitization was enhanced by the activation of small-diameter primary nociceptors (Aβ fibers), allowing low-intensity stimuli to be perceived as pain and causing long-term changes in the spinal neurons.

A study by O’Neill et al.,^(28)^ assessed generalized hyperalgesia in individuals with LBP with intervertebral disc herniation. In the algometry assessment, patients with LBP had a lower PPT than the Control Group.

Furthermore, in a study by Gomes et al.,^(29)^ healthy individuals were assessed before, immediately after, and 20 and 60 minutes after TENS application. They were divided into four groups received different frequencies of electrical stimulation (TG1 - 0Hz, TG2 - 7Hz, TG3 - 100Hz, and TG4 - 255Hz) and a PG. The hypothenar region was the point used for algometry analysis and the elbow for the electrical stimulation. There were no significant differences at any point of assessment.

A trial with 240 healthy subjects assessed the effect of TENS with different frequencies (4-110Hz), intensities (varying between the “tolerable” and “strong but comfortable”), and site of application (radial nerve, gallbladder point B34, or both). The pulse duration was fixed at 200µs with an application duration of 30 minutes. The algometer was applied in the first dorsal interosseous muscle. The patients were assessed immediately before the application of the intervention, during the application (every 10 minutes), immediately after the application of the intervention, and at every 10 minutes for the first 60 minutes after the intervention.

Initially, there was an increase in the PPT when low-frequency stimulation was applied with high intensity maintained for 30 minutes after the stimulation. In contrast, immediate improvement was noted with high-frequency high-intensity stimulation; however, the improvement was not maintained.^(30)^ Therefore, an increase in the PPT caused by the analgesic effects of TENS may be possibly observed, indicating pain improvement.

The main strength of our study is the immediate decrease in the intensity of LBP on application of TENS, which has great relevance in the physical therapists clinical practice. Randomization of participants and comparison with the PG increase the quality of the study and reinforce its applicability. One of the limitations of the study is the lack of assessment after 24 or 48 hours following a single application of the current to verify its effectiveness for pain reduction, and other is that we do not use the Global Perceived Effect Scale that was used to assess the patients’ global perception of improvement and which is important to evaluate the improvements after a physiotherapy program.^(31)^

## CONCLUSION

The two modes of transcutaneous electrical nerve stimulation application, conventional and burst, were effective to modulate pain and increase pain threshold pressure among individuals with chronic low back pain. However, no significant results were found to indicate the best treatment for chronic low back pain.
