# Supplementary material for: Immediate analgesic effect of two modes of transcutaneous electrical nerve stimulation on patients with chronic low back pain: a randomized controlled trial
Source: Einstein (Sao Paulo). 2021 Dec 3;19:eAO6027. doi: 10.31744/einstein_journal/2021AO6027 (PMC8664285; doi:10.31744/einstein_journal/2021AO6027)
Supplement: Translation

# Translation: Efeito analgésico imediato de dois modos da estimulação elétrica transcutânea em indivíduos com dor lombar crônica: ensaio clínico controlado e randomizado

**Authors:** Madeline Luiza Ferreira Pivovarsky, Fernanda Gaideski, Rafael Michel de Macedo, Raciele Ivandra Guarda Korelo, Luiz César Guarita-Souza, Richard Eloin Liebano, Ana Carolina Brandt de Macedo

**Affiliations:** 1 Brasil Universidade Federal do Paraná, Curitiba, PR, Brasil.; 2 Brasil Pontifícia Universidade Católica do Paraná, Curitiba, PR, Brasil.; 3 Brasil Universidade Federal de São Carlos, São Carlos, SP, Brasil.

**Keywords:** Estimulação elétrica nervosa transcutânea, Dor lombar, Medição da dor, Dor crônica, Modalidades de fisioterapia

## Abstract

**Objetivo:**

Comparar e avaliar o efeito analgésico da estimulação elétrica nervosa transcutânea convencional e do *burst* em pacientes com dor lombar crônica.

**Métodos:**

Ensaio clínico controlado, randomizado, três braços e cego. Um total de 105 pacientes com dor lombar crônica não específica, com idade entre 18 a 85 anos foram distribuídos randomicamente nos seguintes grupos: Grupo Placebo (estimulação elétrica simulada), estimulação elétrica nervosa transcutânea convencional (estimulação contínua de 100Hz, 100µs, intensidade sensorial) e estimulação elétrica nervosa transcutânea *burst* (estimulação de 100Hz modulada a 2Hz, 100µs, nível motor). Todos os grupos receberam uma única aplicação da estimulação elétrica nervosa transcutânea por 30 minutos. Os desfechos nomeados intensidade da dor, qualidade da dor e limiar de pressão doloroso foram mensurados pela escala numérica da dor, questionário de McGill e algometria. Os pacientes foram avaliados antes e imediatamente após a aplicação da estimulação elétrica nervosa transcutânea.

**Resultados:**

A intensidade e qualidade da dor melhoraram significativamente (p<0,05) nos Grupos Intervenção. Efeito positivo foi observado nos Grupos Intervenção comparado ao placebo em todos os domínios do questionário de McGill (p<0,05), exceto na intensidade da dor. O limiar de dor a pressão aumentou significativamente (p<0,05) em ambos os Grupos de Intervenção mas não no Grupo Placebo. Contudo, não foi encontrada diferença significativa nas avaliações comparando ambos os Grupos de Intervenção.

**Conclusão:**

Ambos os modos da estimulação elétrica nervosa transcutânea foram efetivos na modulação da dor. Além disso, houve um aumento no limiar de dor a pressão. Não foi encontrado nenhum resultado significativo indicando melhor tratamento para dor lombar crônica.

## INTRODUÇÃO

A divisão de ortopedia da Associação Americana de Fisioterapia define dor lombar crônica (DLC) como dor lombar generalizada com duração de mais de 3 meses consecutivos.^(1)^ O conceito de DLC não específica é geralmente utilizado para descrever esta condição devido a falta de entendimento dos mecanismos da dor. As orientações clínicas^(2)^ definem tais mecanismos como a ausência de sintomas de bandeira vermelha para causas mais graves de dor com patogênese multifactorial.

De acordo com o Anuário Estatístico Brasileiro de Acidentes do Trabalho,^(3)^ aproximadamente 34.253 casos de dorsalgia (CID10 M54) foram registrados no Brasil em 2013, sendo a dor lombar (DL) classificada como a quarta lesão mais comum. Ainda, a lombalgia é indicada como tipo de DL mais frequente, e uma das principais causas de ausência ao trabalho. Além disso, uma revisão sistemática sobre a prevalência de DL no Brasil relatou elevada taxa de prevalência de 1 ano (>50%) desta condição entre adultos.^(4)^

As admissões por DL sobrecarregam todos os serviços de saúde, sendo que várias intervenções têm sido estabelecidas para tratamento da lesão, tais como medicação, cirurgia, educação do paciente, terapia comportamental e fisioterapia.^(2)^ Entre os possíveis tratamentos fisioterapêuticos, os agentes eletrofísicos, tais como a estimulação eléctrica transcutânea do nervo (TENS), são amplamente utilizados no manejo da DL para complementar outras intervenções terapêuticas, especialmente a prática de exercícios. A estimulação do nervo elétrico transcutâneo é relativamente segura, não invasiva, fácil de aplicar^(5)^ e promove alívio da dor durante o exercício.^(2)^ Diferentes modalidades de TENS variando em termos de frequência, amplitude, duração do pulso e forma de onda podem ser utilizadas na prática clínica, tais como os modos convencional ou *burst*.

Estudos têm relatado que a TENS pode reduzir a dor aguda e crônica de diferentes etiologias,^(2,6-10)^ contudo, os parâmetros ideais de tratamento DLC ainda não são conhecidos.^(5)^ Quando a TENS é aplicada com alta frequência, tal como no modo convencional, a intenção fisiológica é produzir Aβ um efeito de despolarização da fibra capaz de inibir a transmissão de informação nociceptiva para a medula espinal.^(6)^ Quando a TENS é aplicada com baixa frequência e por meio de fortes estímulos, tais como o modo de ruptura, a intenção fisiológica é despolarizar as fibras de dor rápida (Aδ) capazes de ativar a analgesia descendente.^(10)^ Além disso, ambas as TENSs ativam receptores opioides no sistema nervoso central que induzem a analgesia, por exemplo, de receptores de serotonina e de Mµ ou delta opioide, quando TENS de baixa ou alta frequência é aplicada.^(7,10)^

Apesar da sua ampla utilização, ainda não existe consenso sobre a real eficácia das TENSs em indivíduos com DL.^(8)^ Uma revisão sistemática^(8)^ sobre a eficácia das TENSs em DLC incluindo quatro ensaios clínicos controlados randomizados de alta qualidade (585 pacientes) em que observou-se alívio da dor em apenas três pacientes em comparação ao Grupo Placebo (GP). Devido aos poucos estudos, não foi possível encontrar evidências sobre a eficácia deste procedimento em pacientes com DL. Consequentemente, não foram encontradas indicativos sobre os parâmetros e modos de estimulação adequados. Jauregui et al.,^(9)^ conduziram metanálise e concluíram que o tratamento com a TENS resultou não só na redução da dor, mas também na redução do uso de medicamentos entre pacientes com DLC tratados por menos de 5 meses.

Johnson et al.,^(10)^ avaliaram o uso de TENS no tratamento da dor aguda causada por múltiplas etiologias em revisão incluindo 19 estudos (1.346 pacientes). Os autores conseguiram avaliar o efeito analgésico da TENS em seis estudos e observaram que a TENS era 3 vezes mais eficaz do que o placebo. No entanto, encontraram evidência baixa a moderada devido a pequena amostra de pacientes e elevado risco de viés nos resultados. Em editorial Johnson et al.,^(11)^ sugeriram que a maioria dos estudos sobre a utilização de TENS eram inconclusivos e de qualidade insuficiente. É importante mencionar que a metanálise de Resende et al.,^(5)^ relatou a necessidade de mais estudos qualitativos sobre os efeitos das TENS em paciente com DLC, que considerem fatores como intensidade da estimulação, dor, avaliação da incapacidade e funcionalidade, e tempo da avaliação dos resultados.

Todas as revisões sistemáticas^(8-10)^ apontam para estudos que compararam único parâmetro da TENS com o GP ou com outras terapias. Dos estudos citados nessas revisões, apenas um^(12)^ comparou três tipos de TENS (TENS convencional, TENS acupuntura, TENS bifásica e GP) ao longo de um mês de tratamento e não foram encontradas diferenças entre os grupos. Contudo, esse estudo não descreveu os parâmetros exatos de aplicação. Há escassez na literatura de estudos que comparem diferentes parâmetros de aplicação das TENSs em DLC.

Embora existam vários estudos sobre o efeito da TENS na DL,^(13,14)^ a maioria deles apenas relata os efeitos de longo prazo. Contudo, o principal objetivo terapêutico durante uma sessão de fisioterapia é alcançar efeito analgésico imediato – tanto para permitir aos pacientes a prática de exercícios físicos quanto para aliviar a dor após a sessão terapêutica.

## OBJETIVO

Comparar e avaliar o efeito analgésico da estimulação elétrica nervosa transcutânea convencional e do burst em pacientes com dor lombar crônica.

## MÉTODOS

### Desenho do estudo

Foi conduzido ensaio controlado, randomizado, de três braços em um único centro. O estudo foi aprovado pelo Comitê de Ética em Pesquisa, CAAE: 44642615.2.0000.0102 e número 1.145.540, e registrado prospectivamente em ensaiosclinicos.gov.br (RBR-59YGR8). Todos os pacientes assinaram o termo de consentimento antes de iniciar o estudo.

### Local do estudo

Estudo realizado no laboratório de reabilitação da Universidade Federal do Paraná (UFPR) e ambulatório do Hospital das Clínicas, Curitiba, PR, Brasil.

### Critério de elegibilidade

Os critérios de inclusão permitiram o ingresso de pacientes com idade entre 18 e 85 anos, com diagnóstico primário consistente de DL não específica (DL não especificada com duração de não mais de 3 meses),^(2)^ sem irradiação e com intensidade de dor mínima de três na Escala Numérica de Dor (END).^(15)^

Os critérios de exclusão compreenderam paciente com causa de dor específica (por exemplo, hérnia de disco, estenose espinhal, fratura vertebral, infecção, tumor lombar associado, osteoporose, artrite reumatóide, processo inflamatório, gravidez, e problemas renais); cirurgia anterior na região lombar, lesões na pele, presença de marcapasso, utilizando medicamento para melhora da DL 48 horas antes da intervenção, e àqueles durante a realização do estavam realizando algum tipo de tratamento para DL.

### Randomização e intervenções

Após a avaliação por linha de base, os pacientes foram randomizados utilizando técnica de bloco de randomização.^(16)^ Cada bloco continha 15 participantes distribuídos randomicamente: GP (n=5), Grupo TENS Convencional (GTC) (n=5) e Grupo TENS *Burst* (GTB) (n=5). Foram inseridos sete blocos, totalizando 105 participantes eram “cegos” a intervenção. O fisioterapeuta que conduziu a intervenção desconhecia os objetivos do estudo.

Estimulação elétrica foi aplicada utilizando o mesmo dispositivo (equipamento TENSys ET 9771, KLD Biosistemas Equipamentos Eletrônicos, São Paulo, SP, Brasil) anteriormente calibrado por MedMart (Brasil). Os participantes foram colocados em posição prona e instruídos para relaxar a musculatura enquanto os procedimentos eram realizados. Quatro eletrodos retangulares de superfície (90x50mm) foram bilateralmente posicionados no nível L3 e L5 processo espinhoso (30mm lateralmente). Com posicionamento quadripolar, a corrente em cada canal cruzava a área dolorosa. Após limpeza da pele com álcool 70%, eletrodos de borracha de silicone impregnada com carbono e flexíveis foram posicionado com fita adesiva e utilizando gel eletrocondutor.^(17)^ O GTC foi administrado utilizando uma simulação contínua (formato de onda bifásica, retangular, simétrica e equilibrada) em alta frequência (100Hz) com duração da pulsação (100µs) e intensidade sensorial. O GTB (TENS acupuntura) foi administrado adotando 100Hz modulado em 2Hz (modo *burst*) e com duração da pulsação de 100μs com intensidade de nível motor. O TENS foi aplicado por apenas 30 minutos, e reavaliação foi feita imediatamente após a intervenção. O tempo de intervalo entre avaliação e reavaliação foi de 30 minutos. Os procedimentos para GP foram similares aqueles de outros grupos, porém a amplitude/intensidade atual não foi aumentada. Os pacientes foram informados que poderiam ou não sentir qualquer sensação no local da aplicação dos eletrodos.^(18)^

### Avaliação e instrumentos

Um fisioterapeuta treinado examinou de forma cega todos os participantes antes e imediatamente após a intervenção. Os resultados medidos foram dor intensa, qualidade da dor, e limiar de dor à pressão (LDP).

### Medição da intensidade da dor com Escala Numérica de Dor

Os participantes foram instruídos a fazer marcação no ponto que melhor representava a intensidade das dores utilizando linha reta de 10cm na END (0=sem dor, 10=pior dor possível).^(19)^

### Qualidade da medição da dor com questionário de dor de McGill

A versão brasileira^(20)^ do questionário de dor de McGill (MPQ) para língua portuguesa contém 78 descrições dividas em 4 subclasses de qualidade de dor (sensorial, afetiva, cognitiva, e miscelâneas). Os descritores foram divididos em 20 subcategorias, cada uma contendo de 4 a 6 palavras. Os pacientes que tinham de escolher uma ou nenhuma das palavras em todas as subcategorias, e o índice numérico foi calculado por meio da soma do número de opções escolhidas, com valor máximo de 20. A versão brasileira do MPQ foi adaptada transculturalmente para o português brasileiro^(18)^ e clinimetricamente testada em estudos anteriores.^(21)^

### Medidas do limiar de dor à pressão com algometria

O LDP foi medido por meio de algômetro (sistema EMG, São José dos Campos, SP, Brasil) com a ponta medindo 1cm^2^ de diâmetro e uma taxa de aplicação de 0,5kgf/cm^2^/s.

Os resultados da algometria de pressão tem mostrado boa validade.^(22)^ Porém, em nosso estudo, uma fisioterapeuta conduziu a avaliação preliminar de confiabilidade entre avaliadores. Essa professional avaliou 10 indivíduos para medir confiabilidade intrateste, com um intervalo de pelo menos 48 horas. A análise intrateste (ITT) indicou confiabilidade excelente para resultados da intervenção (ITT=0,99).

O LDP foi medido bilateralmente nos pontos^(18)^ previamente marcados com lápis dermatográfico: 5cm do processo espinhoso para esquerda e direita da vertebra L3 e L5. Além disso, outros dois pontos foram utilizados como controle e marcados 5cm lateral a tuberosidade da tíbia à esquerda e direita do músculo tibial anterior. Após marcação dos pontos, a ponta do algômetro foi posicionada perperdincular a pele e pressionada gradualmente em taxa de 0,5kgf/cm^2^/s, iniciando com zero kgf/cm^2^/s. Os participantes foram instruídos para falar “pare” quando a pressão com a ponta emborrachada do algômetro causasse dor. Após, este valor foi registrado por pesquisadores e representava o LDP do momento. Após 1 minuto, duas medições adicionais foram realizadas em cada ponto, sendo a média calculada para cada um dos pontos.

### Análise estatística

Todas as análises estatísticas foram realizadas utilizando o SPSS versão 25.0. Os dados foram apresentados como médias±desvio padrão da média e estavam sujeitos a análise de esfericidade e homocedasticidade da variação por meio dos testes de Mauchly e Levene, respectivamente. O intervalo de confiança foi estabelecido em 95% para todas as análises. A avaliação das diferenças entre e dentre os grupos e análise de variância com medidas repetidas foram utilizadas para estimar os valores paramétricos. Para os dados não-paramétricos, o teste de rank Wilcoxon assinado foi adotado para o teste de diferença entre os grupos, e o teste de Kruskal-Walllis para diferenças entre os grupos. O nível de significância foi estabelecido em p<0,05.

### Cálculo da amostra

Empregou-se o programa GPower 3.0 para calcular o tamanho da amostra. A significância estatística foi estabelecida em 0,95, tamanho de efeito moderado (0,35),^(23)^ significância estatística de 95% (1β erro de probabilidade), erro α nível de probabilidade de 0,05 e amostra possível de perda de até 5%. Consequentemente, 35 pacientes por grupo (105 no total) foram necessários.

## RESULTADOS

Um total de 105 pacientes foram avaliados entre agosto de 2015 e agosto de 2017. Nenhum participante desistiu de integrar o estudo. O diagrama dos padrões de consolidação do relato de ensaio é mostrado na figura 1 e as características sociodemográficas apresentadas na tabela 1.

**Figura 1:** Desenho do estudo F: frequência; T: tempo de duração da pulsação; GTC: Grupo TENS Convencional; GTB: Grupo TENS *Burst*; PG: Grupo Placebo.

**Tabela 1:** Características sociodemográficas

Características sociodemográficas	GP (n=35)	GTC (n=35)	GTB (n=35)	Valor de p
Idade [média (DP)]	40,8±2,7	44±2,2	42,6±2,2	0,44
Gênero, n (%)				0,29
Feminino	24 (68,6)	23 (65,7)	27 (77,1)	
Masculino	11 (31,4)	12 (34,3)	8 (22,9)	
Escolaridade, n (%)				0,41
Fundamental incompleto	6 (17,1)	3 (8,6)	7 (20)	
Fundamental completo	2 (5,7)	2 (5,7)	4 (11,4)	
Ensino médio incompleto	2 (5,7)	2 (5,7)	4 (11,4)	
Ensino médio completo	10 (28,6)	6 (17,1)	6 (17,1)	
Ensino superior incompleto	5 (14,3)	5 (14,3)	4 (11,4)	
Ensino superior completo	10 (28,6)	17 (48,6)	10 (28,6)	
Hábitos, n (%)				
Tabagismo	4 (11,4)	4 (11,4)	2 (5,7)	0,42
Consumo de álcool	1 (2,9)	0 (0)	1 (2,9)	0,38
Sedentarismo	27 (77,1)	22 (62,9)	23 (65,7)	0,25
Tempo de dor em anos (média, mínimo, máximo, mediana)	6,81; 0,5; 30; 5	8,8; 1; 30; 7	7,25; 0,5; 30; 3	0,31
Local da dor, n (%)				0,94
Centro	7 (20)	9 (25,7)	9 (25,7)	
Direita	7 (20)	6 (17,1)	7 (20)	
Esquerda	6 (17,1)	8 (22,9)	6 (17,1)	
Bilateral	15 (42,9)	12 (34,3)	13 (37,1)	
Período de piora da dor, n (%)				0,06
Manhã	9 (25,7)	11 (31,4)	4 (11,4)	
Tarde	11 (31,4)	7 (10)	19 (54,3)	
Noite	15 (42,9)	17 (48,6)	12 (34,3)	
Atividades acionadoras de piora da dor, n (%)				
Caminhar	11 (31,4)	11 (31,4)	14 (40)	0,75
Sentar	8 (22,9)	10 (28,6)	11 (31,4)	0,79
Curvar	19 (54,3)	21 (60)	15 (42,9)	0,15
Levantar	13 (37,1)	12 (34,3)	11 (31,4)	0,80
Subir escadas	11 (31,4)	7 (20)	6 (17,1)	0,77
Esforço/ Levantar objetos	29 (82,9)	31 (88,6)	29 (82,9)	0,51

GP: Grupo Placebo; GTC: Grupo TENS Convencional; GTB: Grupo TENS *Burst*; DP: desvio padrão.

A tabela 2 mostra a média e desvio padrão da intensidade da dor END e qualidade da dor (escore MPQ). Uma diferença estaticamente significante foi notada nos parâmetros dentro dos grupos.

**Tabela 2:** Média (desvio padrão) na linha de base (antes) e 30 minutos (após) seguimento de indivíduos com dor lombar crônica que recebeu Estimulação Elétrica Nervosa Transcutânea na modalidade convencional, estimulação elétrica nervosa transcutânea na modalidade convencional, estimulação elétrica nervosa transcutânea *burst* ou Grupo Placebo

Resultado	Intervenções					

	GTC (n=35) Média (DP)		GTB (n=35) Média (DP)		GP (n=35) Média (DP)	

	Antes	Depois	Antes	Depois	Antes	Depois
Dor (0-10)	5,4 (1,6)	2,3 (2,1)*	4,7 (2,2)	2,4 (2,1)*	4,2 (2,2)	3,0 (2,4)*
MPQ						
Sensorial	7,1 (2,2)	3,8 (3,4)*	7,8 (2,3)	3,4 (3,4)*	7,5 (2,4)	4,6 (3,4)*
Afetivo	3,0 (1,8)	0,6 (1,3)*	3,7 (1,4)	1,1 (1,5)*	3,3 (1,3)	1,6 (1,6)*
Cognitivo	1,0 (0,1)	0,5 (0,5)*	1,0 (0)	0,4 (0,5)*	0,9 (0,1)	0,6 (0,4)*
Miscelâneas	2,5 (1,1)	1,3 (1,4)*	3,0 (0,8)	1,4 (1,5)*	2,8 (1,3)	1,9 (1,5)*
Total	13,5 (5,1)	6,3 (5,8)*	15,5 (4,1)	6,6 (6,3)*	14,5 (5,1)	8,8 (6,2)*

* p<0,05 entre grupos.

DP: desvio padrão; GP: Grupo Placebo; GTC: Grupo TENS Convencional; GTB: Grupo TENS *Burst*; MPQ: Questionário da dor de McGill.

A figura 2 mostra os resultados LDP. O grupo TENS mostrou um aumento significante aumento no LDP (p<0,05) em todos pontos lombares na algômetria de pressão. Em oposição, foi observada uma redução não significante no GP.

**Figura 2:** Limiar de pressão de dor na linha de base (antes) e 30 minutos (após) seguimento de indivíduos com dor crônica lombar que receberam estimulação elétrica nervosa transcutânea na modalidade convencional e estimulação elétrica nervosa transcutânea *burst* ou Grupo Placebo * p<0,05 entre grupos. LL3: terceira vertebra lombar esquerda; LR3: terceira vertebra lombar direita; LL5: quinta vertebra lombar esquerda; LR5: quinta vertebra lombar direita; GP: Grupo Placebo; GTC: Grupo TENS Convencional; GTB: Grupo TENS *Burst*.

A tabela 3 mostra a análise entre grupos analisados para todas as comparações. A análise estatística não mostrou diferenças entre Grupos Intervenção (GTC e GTB) por meio da Escala Visual Análoga (EVA) comparado ao placebo. Além disso, enquanto uma redução de 28,5% na intensidade da dor foi notada no GP, uma interversão de 30 minutos levou a redução de 57,4% e 48,9% na intensidade da dor em GTC e GTB, respectivamente. Uma diferença estatisticamente significante foi observada entre o Grupo TENs e GP baseada nas subcategorias MPQ (sensorial, afetiva, cognitiva, e miscelâneas), e o índice numérico. Em MPQ, a GTC mostrou aumento de 53,3% no índice numérico de 57,4% no GTB, enquanto no GP foi de apenas 39,3%. Porém, não foi observado diferença estatística quando comparados tanto o GTC quanto o GTB. Na algometria de pressão, não se observou diferença estatística na comparação entre GTC e GTB com a GP, apesar de um aumento no limiar de pressão de dor ter sido encontrado nos Grupos Intervenção e redução em placebo.

**Tabela 3:** Diferença entre grupo de seguimento 30 minutos após randomização para indivíduos com dor lombar crônica que receberam estimulação elétrica nervosa transcutânea na modalidade convencional, estimulação elétrica nervosa transcutânea *burst* Grupo Placebo

Resultado	GTC *versus* Placebo	Valor de p	GTB *versus* Placebo	Valor de p	GTC *versus* GTB	Valor de p
Dor (0-10)	0,1 (-0,9-1,2)	1,00	0,2 (-0,9-1,2)	1,00	0,3 (-0,7-1,4)	1,00
MPQ						
Sensorial (0-10)	2,1 (1,1-3,1)	0,00*	1,9 (0,9-2,9)	0,00*	0,2 (-0,7-1,1)	1,00
Afetivo (0-5)	0,9 (0,3-1,4)	0,00*	0,8 (0,2-1,4)	0,02*	-0,1 (-0,4-0,6)	1,00
Cognitivo (0-1)	0,2 (0,1-0,3)	0,00*	0,2 (0,1-0,3)	0,00*	1,8 (-013-0,1)	1,00
Miscelâneas (0-4)	1,0 (0,5-1,4)	0,00*	0,9 (0,4-1,3)	0,00*	-0,8 (-0,3-0,5)	0,18
Total (0-20)	1,7 (3,8-1,4)	0,03*	-0,5 (3,2-2,0)	0,02*	-1,1 (-1,5-3,7)	0,80
Algômetria						
LTA	-0,9 (-1,1-0,9)	1,00	-0,1 (-1,1-0,9)	1,00	-0,2 (-1,2-0,9)	1,00
RTA	0,0 (-1,1-1,1)	1,00	-0,1 (-1,2-0,9)	1,00	-0,15 (-1,2-0,9)	1,00
LL3	0,2 (-0,7-1,2)	1,00	0,0 (-0,9-0,0)	1,00	-0,2 (-1,1-0,7)	1,00
RL3	0,2 (-0,7-0,5)	1,00	0,0 (-0,9-0,9)	1,00	0,2 (-1,1-0,7)	1,00
LL5	0,2 (-0,8-1,2)	1,00	0,0 (-1,0-1,0)	1,00	-0,2 (-1,2-0,8)	1,00
RL5	0,2 (-0,7-1,2)	1,00	-0,1 (-1,0-0,8)	1,00	-0,3 (-1,3-0,6)	1,00

* Diferença significante (p<0,05). Diferença em média ajustada (IC95%). Seguimento de linha de base por 30 minutos (IC95%).

IC95%: intervalo de confiança de 95%; RTA: músculo tibial anterior à direita; LTA: músculo tibial anterior à esquerda; LL3: terceira vértebra lombar à esquerda; RL3: terceira vértebra lombar à direita; LL5: quinta vértebra lombar à esquerda; RL5: quinta vértebra lombar à direita; GTC: Grupo TENS Convencional; GTB: Grupo TENS *Burst*; MPQ: questionário de dor de McGill.

## DISCUSSÃO

Este estudo avaliou os efeitos analgésicos imediatos dos modos TENS Convencional e *Burst* em pacientes com DLC avaliados que utilizaram END, MPQ, e algometria de pressão.

Pelo conhecimento dos autores, atualmente este é o primeiro estudo da que comparou os efeitos analgésicos imediatos de dois modos diferentes de TENS em DLC. O alívio imediato da dor é necessário para permitir aos pacientes a realizar exercícios de reabilitação, já que a dor pode ser um fator limitante ou intenso que pode ocorrer durante a sessão de terapia.

Neste estudo, não identificou-se diferença significativa nos resultados da END entre os Grupos TENS. Portanto, os modos TENS Convencional e *Burst* foram igualmente efetivos em providenciar alívio imediato da dor. O TENS de alta e baixa frequência (convencional) promove analgesia por meio da liberação de opioides endógenos no nível espinhal e medula rostral ventral. As frequências de diferentes estímulos levaram a liberação de opiodes diferentes, ou seja, alta frequências (maior de 50Hz) estímulo de receptores δ-opiodes e baixa frequências (menor de 10Hz) receptores de µ-opioide ativo.^(24)^ Considerando que os caminhos analgésicos não são os mesmos, esperou-se que as respostas também poderiam ser distintas. Contudo, tal distinção não foi observada.^(9)^ Esses dados corroboram com estudo conduzido por Cheing et al.,^(25)^ em que TENS (80Hz, 140μs, 60 minutos) foi aplicado em indivíduos com dor crônica e redução do padrão da dor verificado por meio do escore EVA após aplicação única. Tousignant-Laflamme et al.,^(26)^ comparou os resultados da 1ª rodada de aplicação de TENS acupuntura em períodos com tempos diferentes (15 e 30 minutos) em 11 pacientes com DLC e não encontrou diferenças no escore VAS entre os grupos. Porém, não houve comparação com GP.

Na avaliação por algometria por pressão, os resultados positivos foram observados, com um aumento no LPD variando de 8% para 15% em grupos tratrados com TENS comparado com uma redução de 2% a 5% no GP. Ebaldi et al.,^(1)^ avaliou os efeitos analgásicos de TENS (120HZ, 100μs, 15 minutos) e correntes dinâmicas no LDP no Grupo TENS. Em um ensaio conduzido por Giesbrecht et al.,^(27)^ o LDP em pacientes com DL foi comparado ao de pacientes saudáveis. Os resultados revelaram que o grupo com DL teve menor LDL, que indica sensibilidade no local. De acordo com estudo, a sensibilização central foi melhorado por ativação de nociceptores primários de menor diâmetro (fibras Aβ), permitindo baixa-intensidade de estimulo para ser percebido como dor e causa de mudanças de curta duração em neurônios espinhais.

Um estudo de O’Neil et al.,^(28)^ avaliou hiperalgesia generalizada em indivíduos com DL com herniação de disco intervertebral. Na avaliação de algometria os pacientes com DL apresentaram LDP menor do que o Grupo Controle.

Além disso, no estudo de Gomes et al.,^(29)^ indivíduos saudáveis foram avaliados antes, imediatamente após, e de 20 a 30 minutos após a aplicação de TENS. Os participantes foram divididos em grupos de quatro que receberam frequências diferentes de estímulo elétrico (TG1 - 0Hz, TG2 - 7Hz, TG3 - 100Hz, eTG4 - 255Hz) e um GP. A região hipotênar foi o ponto utilizado por análise de algometria e cotovelo por estimulação elétrica. Não observou-se diferenças significativas em qualquer um dos pontos de avaliação.

Um ensaio de 240 indivíduos saudáveis avaliou os efeitos de TENS com diferentes frequências (4-110Hz), intensidades (variando entre “tolerável” e “intensa, mas confortável”), e local de aplicação (nervo radial, ponto B34, ou ambos). A duração de pulso foi fixada em 200µs com uma aplicação de duração de 30 minutos. O algômetro foi aplicado no primeiro interósseo dorsal. Os pacientes foram avaliados imediatamente anterior a aplicação da intervenção, durante a aplicação (cada 10 minutos), imediatamente após a aplicação da intervenção, e cada 10 minutos ao longo dos primeiros 60 minutos após a intervenção.

Inicialmente, houve um aumento no LDP quando estímulo de baixa frequência foi aplicado com alta intensidade e mantida por cerca de 30 minutos após o estímulo. Em posição, a melhora imediata foi notada com alta frequência com estimulo de alta intensidade; porém, a melhora não foi mantida.^(30)^ Portanto, um aumento de LDP causado por efeitos analgésicos de TENS pode ser possivelmente observado, indicando melhora da dor.

O principal ponto forte deste estudo foi a redução imediata na intensidade do DL nas aplicações de TENS, que foi de maior relevância na prática clínica de terapeutas físicos. A randomização dos participantes e comparações com GP aumentou a qualidade do estudo e reforçou sua aplicabilidade. Uma das limitações do nosso estudo é a falta de avaliação 24 ou 48 horas após a aplicação única de corrente para verificar sua efetividade para redução de dor e outra questão é a não adoção da Escala de Percepção do Efeito Global que foi utilizada para avaliar a percepção global de melhoria dos pacientes e foi importante para avaliar as melhorias após o programa de fisioterapia.^(31)^

## CONCLUSÃO

Os dois modos de aplicação de estimulação elétrica nervosa transcutânea convencional ou *burst* foram efetivos para modular a dor e aumentar o limiar de dor à pressão entre indivíduos com dor lombar crônica. Contudo, não se encontrou resultados significantes para indicar o melhor tratamento para dor lombar crônica.
